# A Telerehabilitation Program for Maintaining Functional Capacity in Patients With Chronic Lung Diseases During a Period of COVID-19 Social Isolation: Quasi-Experimental Retrospective Study

**DOI:** 10.2196/40094

**Published:** 2022-12-22

**Authors:** Aline Paula Miozzo, Natiele Camponogara Righi, Maria Luiza Yumi Shizukuishi, Hérica Marques Ferreira Aguilar, Juliessa Florian, Scheila da Costa Machado, Jociane Schardong, Rodrigo Della Méa Plentz

**Affiliations:** 1 Physiotherapy Department Universidade Federal de Ciências da Saúde de Porto Alegre Porto Alegre Brazil; 2 Physiotherapy Department Irmandade Santa Casa de Misericórdia de Porto Alegre Porto Alegre Brazil

**Keywords:** telerehabilitation, lung diseases, social isolation, COVID-19, pulmonary rehabilitation, pulmonary, rehabilitation, quality of life, chronic disease, mental health, social functioning, patient outcome

## Abstract

**Background:**

Pulmonary diseases represent a great cause of disability and mortality in the world, and given the progression of these pathologies, pulmonary rehabilitation programs have proven to be effective for people with chronic respiratory diseases. During the COVID-19 pandemic, telerehabilitation has become an alternative for patients with such diseases.

**Objective:**

The aim of this study was to compare the outcomes (ie, functional capacity and quality of life) of telerehabilitation to those of usual care among patients who previously participated in face-to-face pulmonary rehabilitation programs.

**Methods:**

We conducted a quasi-experimental retrospective study from April 2020 to August 2021. A total of 32 patients with chronic lung diseases were included and divided into the control and intervention groups. The intervention group performed telerehabilitation synchronously twice per week and was supervised by a physical therapist during breathing, strengthening, and aerobic exercises. Changes in the degree of dyspnea and leg discomfort were assessed based on changes in Borg scale scores. The control group did not perform any activities during the period of social isolation. Functional capacity was assessed with the 6-minute walk test, and quality of life was assessed with the Medical Outcomes Study 36-item Short Form Health Survey.

**Results:**

The telerehabilitation group’s mean 6-minute walk distance decreased by 39 m, while that of the control group decreased by 120 m. There was a difference of 81 m between the groups’ mean 6-minute walk distances (*P*=.02). In relation to the quality of life, telerehabilitation was shown to improve the following two domains: social functioning and mental health.

**Conclusions:**

Telerehabilitation programs for patients with chronic lung diseases can ease the deleterious effects of disease progression, be used to maintain functional capacity, and improve aspects of quality of life.

## Introduction

In 2019, the whole world was impacted by the onset of a new disease—COVID-19. SARS-CoV-2 infection, in the most severe cases, leads to acute respiratory syndrome. Further, it has reached high incidence rates and is associated with mortality, resulting in a pandemic state [1]. The impact of the disease has exceeded expectations with regard to mortality, complications, and hospital costs, leading to significant socioeconomic, political, and psychosocial impacts. Many services that were considered nonessential had their activities stopped, and people were encouraged to seek alternatives to maintain their routines within their own homes or seek other options so that patients with pre-existing diseases could continue to be assisted, thereby avoiding detriments to physical well-being and lung capacity and reducing hospital admissions and mortality rates [2].

Lung diseases, such as the more prevalent chronic obstructive pulmonary disease (COPD) and interstitial lung disease, represent a great worldwide cause of disability and mortality with a growing burden [3-5]. These progressive pathologies can cause dyspnea; fatigue; and reductions in physical activity, exercise tolerance, muscle strength, and health-related quality of life (HRQOL) [6-8]. These may result in immobility, dependency on help from others, and social isolation [4,7].

In this context, given the progression of these pathologies, pulmonary rehabilitation programs (PRPs) are interventions that have proven to be effective for people with chronic respiratory diseases [8-10]. Among the main benefits of PRPs, the improvements in exercise capacity, HRQOL, and survival; the reductions in the intensity of dyspnea, the number of hospitalizations, and the length of hospital stays; and decreased anxiety and depression can be highlighted [11].

Such benefits persist for 8 to 12 weeks among patients with chronic respiratory diseases who participate in face-to-face PRPs [12]. However, over the past 2 years, billions of people have continued to maintain social distancing as a measure for containing the spread of SARS-CoV-2 infection. Social isolation leads to loneliness and chronic boredom, which can have negative effects on the physical and mental well-being of patients with chronic respiratory diseases [2]. Thus, telerehabilitation may offer an alternative for ensuring patients’ participation in PRPs [12]. The term *telerehabilitation* has been used to describe the delivery of rehabilitation by using telecommunication technology [13].

Recent studies show that telerehabilitation for patients with chronic lung diseases, when compared to face-to-face PRPs, allows for the maintenance of important outcomes, such as functional capacity, which is assessed with the 6-minute walk test (6MWT) [8,14-17]. Furthermore, it is safe [8,18], with no increase in the rate of adverse events [12], and it has good patient compliance [14]. Telerehabilitation has also been able to reduce disease exacerbations as well as the mean number and duration of hospitalizations [16,19]. However, there are still no studies on conducting telerehabilitation with these patients during the COVID-19 pandemic.

Since 2010, the World Health Organization has encouraged the use of telemedicine to maximize health services while respecting cultural, demographic, and gender differences [20]. In the field of physiotherapy, World Physiotherapy and the International Network of Physical Therapy Regulatory Authorities (INPTRA) have highlighted that the use of modern technologies and practices through digital means creates an excellent opportunity for physiotherapists to engage with broad audiences to improve effects and impacts, resulting in the provision of services, resources, and information in an easier and faster way [21]. However, in Brazil, the law that defends the use of telemedicine was enacted [22] only because of the pandemic, and the Federal Council of Physiotherapy regulated the permission of non–face-to-face services in 2020. The teleconsultation and telemonitoring modalities have thus become recent practices in physiotherapy [23]. Therefore, the aim of this study was to evaluate and compare the effects of telerehabilitation and usual care on the functional capacity and quality of life of patients with chronic lung diseases who were previous participants of face-to-face PRPs, in the context of the social isolation imposed by the COVID-19 pandemic.

## Methods

This quasi-experimental retrospective study was carried out at the Pulmonary Rehabilitation Service of the Pavilhão Pereira Filho at Irmandade da Santa Casa de Misericórdia de Porto Alegre (ISCMPA), through the institutional portal and on site, from April 2020 to August 2021.

### Ethics Approval

This study was approved by the Ethics Committees in Human Research of ISCMPA and was registered under approval number 04453412.7.0000.5335. All participants signed the informed consent form.

### Study Sample

The telerehabilitation program was structured based on the need to retain the content of its face-to-face form as much as possible during social isolation. The sample was for convenience and included patients who were enrolled in and were undergoing pulmonary rehabilitation at the Pulmonary Rehabilitation Service of the Pavilhão Pereira Filho at ISCMPA before the period of social isolation. Patients were recruited from July 2020 to July 2021. The inclusion criteria were patients diagnosed with COPD, interstitial lung disease, bronchiectasis, or pulmonary emphysema; patients of both sexes; patients aged between 18 and 80 years; and patients on optimized drug therapy. The exclusion criteria were patients who were discontinued from the program; patients undergoing lung transplantation; patients who were hospitalized, resulting in the interruption of the home program; and failure to sign the informed consent form. A total of 32 patients were included—14 in the control group and 18 in the telerehabilitation group.

The physical training component of the face-to-face PRP was administered by 2 physical therapists, with sessions conducted 3 times per week. During this physical training, patients performed breathing exercises (respiratory cycle) as a warm-up, followed by arm and leg exercises for muscle strengthening, which involved an initial load of 30% of the 1-repetition maximum testing load and 1 set of 10 repetitions per exercise. Aerobic exercises were also performed on a treadmill, beginning at 70% of the patients’ speed on the 6MWT, with progression. During the PRP, all patients received continuous oxygen therapy in accordance with their medical prescriptions, and they were constantly monitored via pulse oximetry to maintain an oxygen saturation of ≥92%. The modified Borg scale was used for measuring the degree of dyspnea and leg discomfort [24]. This scale is graded from 0 to 10, where 0 indicates no feeling of shortness of breath or discomfort, and 10 indicates the maximum feeling of shortness of breath or discomfort. The Borg scale seems to be an affordable, practical, and valid tool for monitoring and prescribing exercise intensity, independent of sex, age, exercise modality, and physical activity level [25,26]. Assessments of functional capacity and quality of life were collected every 36 sessions [24].

In March 2020, the face-to-face form of the program had to be closed due to the social isolation measures that were implemented to combat the advance of COVID-19. All patients were invited to follow up remotely, and those who accepted the invitation entered the telerehabilitation program.

### Telerehabilitation Program

Educational materials containing photos and explanations for performing exercises at home were made available at the beginning of social isolation, and phone calls were also made once per week. Subsequently, the institution developed a connection portal via the institutional website, so that the consultations could take place remotely, and physical therapists, residents, and physical therapy interns began to monitor 26 patients via the internet. The telerehabilitation group held 2 synchronous sessions per week and was monitored by physiotherapists through the institutional portal. These physiotherapists also provided guidance on how to perform the exercises once per day every day. Patients performed breathing exercises (deep inspiration and expiration with a labial fret) during sessions, and resistance exercises for the upper and lower limbs, metabolic exercises, and aerobic exercises (ie, walking, treadmill exercises, or ergometric bicycle exercises) were performed according to patients’ availability. Patients were encouraged to buy weights for performing strength exercises, and if this was not possible, the rehabilitation service made the materials available by loaning them to patients. Additionally, pulse oximeters were used to monitor the patients during the exercises. Patients were monitored for heart rate and peripheral oxygen saturation by using a pulse oximeter, and the modified Borg scale was used for measuring the degree of dyspnea and leg discomfort [25,26]. This scale was used due to the familiarity that patients had with its use during the face-to-face PRP. Patients in the control group did not perform any activities during the period of social isolation and therefore did not participate in this phase of the research.

In July 2021, the transition from face-to-face rehabilitation to its hybrid form took place, in which the patients performed a face-to-face session every other week, intercalating with a web-based session. Functional capacity and quality of life assessments were performed at the times when patients returned to the face-to-face portions of the PRP.

### Outcomes

Functional capacity was the primary outcome and was assessed through the 6MWT, in accordance with the recommendations of the American Thoracic Society [27]. The patients made their way through a 30-m corridor (delimited by cones) for 6 minutes, and they were encouraged by the evaluator every minute. Data on heart rate, blood pressure, and effort perception (based on the Borg scale) were obtained before and after the test. In order to evaluate the impact on quality of life and health maintenance, the patients were invited to fill out a questionnaire. The Medical Outcomes Study 36-item Short Form Health Survey (SF-36) [28] was used to evaluate HRQOL. Through this questionnaire, the following eight domains of HRQOL were assessed: physical functioning, physical role, physical pain, general health, vitality, social functioning, emotional role, and mental health [28].

The last assessment in the face-to-face form of the PRP was considered the initial assessment for this study. The final assessment was performed when patients in both groups returned to the face-to-face portion of the PRP. In addition, data, such as the need for continuous oxygen use only during exercise, were collected.

### Statistical Analysis

For the data analysis, SPSS software version 23.0 (IBM Corp) was used. Data normality was verified by using the Kolmogorov-Smirnov test. Continuous data were presented as means and SDs, and categorical data were described as frequencies and percentages. Comparisons were performed by using a *t* test or Mann-Whitney test. The adopted significance level was 5%.

## Results

**Figure 1:**
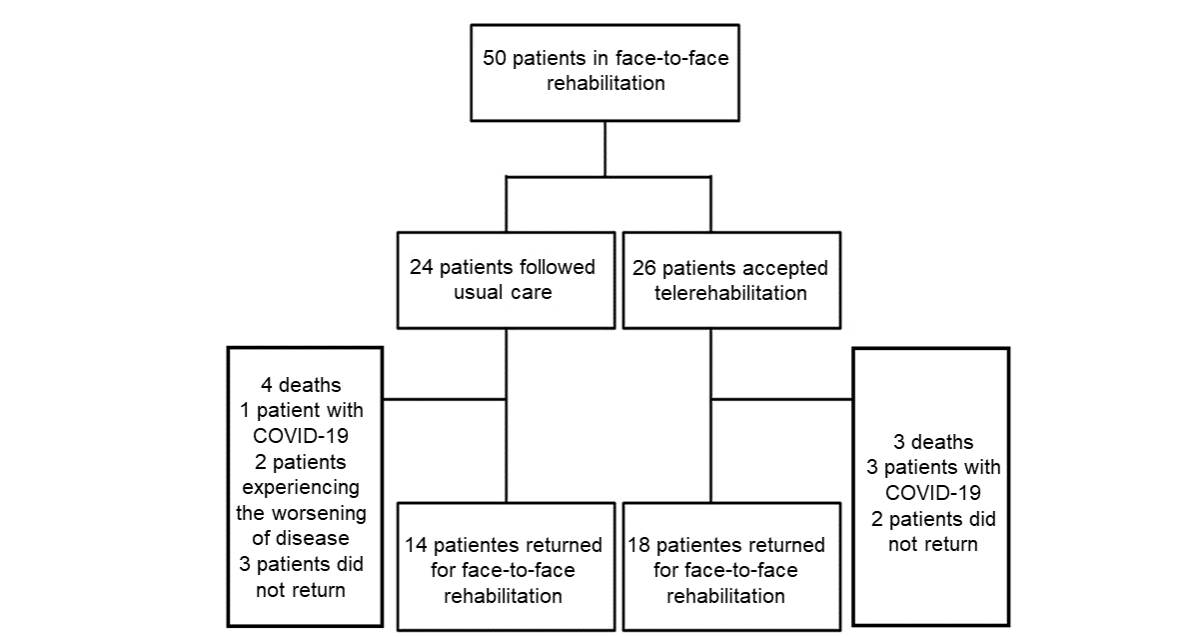
Patient flowchart.

A total of 32 patients were included in the sample—14 in the control group and 18 in the telerehabilitation group. Of note, 63% (20/32) of participants were female, and the control group was older and had higher BMIs when compared to the telerehabilitation group. With regard to lung function, the groups were homogeneous, and the most prevalent pathologies were pulmonary fibrosis, pulmonary emphysema, and COPD. A patient flowchart is shown in Figure 1, and clinical characteristics are presented in Table 1.

**Figure 2:**
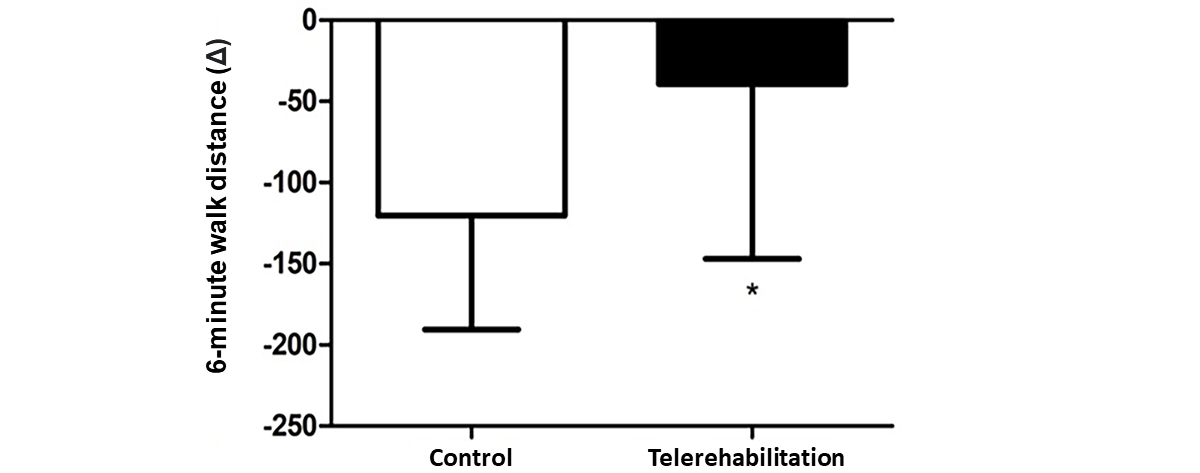
Participants' 6-minute walk test distances. *P=.02.

In relation to the main outcome, prior to the pandemic, the control group walked more than the telerehabilitation group in the 6MWT (mean 465, SD 84 m vs mean 388, SD 121 m). After about 1.5 years of isolation, the control group walked a mean of 344 (SD 92) m, and the telerehabilitation group walked a mean of 348 (SD 146) m. It can be noted that the control group’s mean 6-minute walk distance (6MWD) decreased by 120 m, which represents 4 fewer laps in the 6MWT, and the telerehabilitation group’s mean 6MWD decreased by only 39 m, which represents 1 fewer lap in the test. There was a difference of 81 m between the groups’ mean 6MWDs (*P*=.02; Figure 2).

As for HRQOL, of the 8 SF-36 domains, 6 did not show a significant difference in results (physical functioning: *P*=.95; physical role: *P*=.13; physical pain: *P*=.24; general health: *P*=.92; vitality: *P*=.34; emotional role: *P*=.76). However, for important outcomes, such as social functioning and mental health, telerehabilitation showed a beneficial effect (*P*=.03 and *P*=.02, respectively; Table 2).

The loss of functional capacity among patients with chronic lung diseases due to the pause in rehabilitation activities imposed by COVID-19 social isolation was also observed through a qualitative analysis of data regarding the use of continuous oxygen only during exercise. Of the 18 patients in the telerehabilitation group, only 8 used continuous oxygen to perform the exercises, and after the isolation period, all patients needed continuous oxygen for these activities. In the control group, before isolation, no patients used continuous oxygen to perform the exercises, and after the isolation period, 7 patients started to use it. That is, there was about a 50% increase in the need to use continuous oxygen to perform exercises after the period of social isolation in both groups.

**Table 1:** Clinical characteristics.

Characteristics			Control group (n=14)		Telerehabilitation group (n=18)		*P* value^a^
Sex (male), n			4		8		N/A^b^
Age (years), mean (SD)			73.4 (7.4)		54.5 (8.7)		<.001
BMI (kg/m^2^), mean (SD)			28 (3.9)		23.9 (3.6)		.004
**Pulmonary function (control group: n=12; telerehabilitation group: n=17), mean (SD)**							
	FEV1^c^ (L)	1.2 (0.5)		1.1 (0.7)		.25	
	FVC^d^ (L)	2.1 (0.5)		1.7 (0.7)		.09	
	FEV1 to FVC ratio	54.9 (16.6)		57.5 (21.1)		.73	
**Baseline disease, n (%)**							
	Pulmonary fibrosis	2 (14)		5 (28)		N/A	
	Bronchiectasis	3 (21)		2 (11)		N/A	
	Pulmonary emphysema	2 (14)		4 (22)		N/A	
	Chronic obstructive pulmonary disease	7 (50)		6 (33)		N/A	
	Pulmonary arterial hypertension	0 (0)		1 (6)		N/A	
**Comorbidities, n (%)**							
	Systemic arterial hypertension	6 (43)		5 (28)		N/A	
	Atrial fibrillation	1 (7)		1 (6)		N/A	
	Diabetes	3 (21)		2 (11)		N/A	
	Dyslipidemia	2 (14)		0 (0)		N/A	
	Osteoporosis	1 (7)		1 (6)		N/A	
	Depression	1 (7)		1 (6)		N/A	
	Fibromyalgia	2 (14)		0 (0)		N/A	
	Hypothyroidism	2 (14)		2 (11)		N/A	
	Pulmonary arterial hypertension	0 (0)		2 (11)		N/A	
	Stroke	0 (0)		1 (6)		N/A	

^a^Derived from a *t* test or Mann-Whitney test.

^b^N/A: not applicable.

^c^FEV1: forced expired volume in the first second.

^d^FVC: forced vital capacity.

**Table 2:** Quality of life.

SF-36^a^ domains	Control group scores			Telerehabilitation group scores				*P* value^b^
	Before isolation, mean (SD)	After isolation, mean (SD)	Mean ∆	Before isolation, mean (SD)	After isolation, mean (SD)	Mean ∆		
Physical functioning	38 (26)	32 (28)	−6	20 (18)	15 (16)	−5	.95	
Physical role	45 (48)	32 (36)	−13	25 (36)	28 (35)	3	.13	
Physical pain	53 (17)	50 (20)	−4	46 (12)	49 (13)	3	.24	
General health	49 (22)	44 (23)	−5	38 (24)	33 (22)	−5	.92	
Vitality	56 (23)	45 (17)	−10	49 (17)	44 (15)	−6	.34	
Social functioning	73 (30)	62 (27)	−12	57 (27)	74 (21)	17	.03^c^	
Emotional role	62 (43)	55 (43)	−7	63 (46)	63 (46)	0	.76	
Mental health	75 (18)	64 (20)	−11	72 (14)	74 (14)	2	.02^c^	

^a^SF-36: 36-item Short Form Health Survey.

^b^Derived from a *t* test or Mann-Whitney test.

^c^Significant at the *P*<.05 level.

## Discussion

### Principal Findings

This study proposed to measure the effects of a telerehabilitation program in patients with chronic lung diseases and compare these effects to those of usual care after PRP interruption due to the social isolation imposed by COVID-19. The main findings of this study were the maintenance of functional capacity through telerehabilitation and the improvement in quality of life domains, such as social functioning and mental health. Furthermore, there was an increase in the need to use continuous oxygen during exercise in 44% (8/18) and 50% (7/14) of patients in the telerehabilitation and control groups, respectively.

As chronic lung diseases advance, exertional breathlessness is triggered by simple activities of daily life and is the strongest determinant of functional capacity and HRQOL [13,14]. The importance of showing positive results in the 6MWT is associated with the fact that, in patients with chronic lung diseases, a lower 6MWD is strongly and independently associated with an increased mortality rate and is a better predictor of death at 6 months than forced vital capacity [29-31]. Our study revealed that telerehabilitation resulted in the maintenance of the 6MWDs obtained in the 6MWT, while the control group’s 6MWDs decreased by 4 laps in this test.

Other recent studies that compared telerehabilitation to usual care also found this same maintenance of functional capacity in patients with COPD [16] and in patients with idiopathic pulmonary fibrosis (IPF) [14]. As in our study, Cerdán-de-Las-Heras et al [14] also found a significant reduction in the distances covered in the 6MWT by the control group. When compared to face-to-face rehabilitation, this same maintenance was found in patients with COPD [17,18,32].

In addition to this important benefit, some studies have shown that telerehabilitation can be as effective as face-to-face rehabilitation in reducing the number of exacerbations in patients with chronic lung diseases, as well as reducing the mean duration and number of hospitalizations [16,19]. Telerehabilitation has been shown to be safe and well tolerated among patients with chronic lung diseases, in addition to not being related to an increase in the rate of adverse events [8,12,14,18] and being greatly cost-effective [33].

An important finding of our study is the improvement of the social functioning and mental health components of the SF-36. Corroborating our study, Galdiz et al [15] also found differences in the mental health component when comparing telerehabilitation with face-to-face rehabilitation. Magaluti et al [12] also showed positive results for HRQOL. Cerdán-de-Las-Heras et al [14], on the other hand, did not demonstrate differences in the HRQOL of patients with IPF when comparing telerehabilitation with usual care [14].

It is important to highlight that social isolation leads to loneliness and boredom [2], which result in a reduction in physical activity levels and the worsening of health and general condition [34]. In addition, because of the social isolation imposed by the COVID-19 pandemic, there has been a worsening in life habits, such as increases in the consumption of alcohol and other substances, which directly correlate with mental health factors [35]. Mental health and general well-being have been severely affected by COVID-19 [36]. In March 2020, 25% of the Canadian population reported poor to regular mental health, whereas only 8% reported this degree of mental health in 2018 [37]. Similar results were found in other countries, such as the United Kingdom, Italy, and Spain [38]. In China, half of the adult population experienced symptoms of anxiety and depression [39]. Thus, there is a need for programs that intervene in the quality of life and are related to the mental health and well-being of the population. These programs can be delivered primarily through telecommunication during periods of isolation, such as those that are imposed due to health reasons [40].

Given the abovementioned issues, it is understood that chronic lung diseases result in the progression of a cycle of physical inactivity, low exercise tolerance, increased dyspnea, and the worsening of HRQOL [14]. Additionally, social isolation increases loneliness and contributes to these adverse effects [2], which can explain the increased need for continuous oxygen use during exercise that was found in our study. With regard to the supply of oxygen in patients with chronic lung diseases, a study showed that providing oxygen through a cylinder for home use for 15 days to patients with IPF showed positive 6MWT results regarding the sensation of shortness of breath and the ability to walk [41]. Further, in a review by Bell et al [42], when exercises were performed with an oxygen supply, the groups showed improvements in the physical functioning, social functioning, mental health, and general health domains of the SF-36.

Telerehabilitation is already well documented in the literature. It is an effective, safe practice; promotes the maintenance and improvement of functional capacity and quality of life; and is cost-effective [8,14-19,33]. This practice is already recommended by several institutions, such as the World Health Organization, World Physiotherapy, and the INPTRA, but each country has its legal issues, depending on the profession [20,21]. For physical therapy, our data are important, given that organizations that carry out programs remotely can reach a greater number of patients with the most diverse comorbidities, even those who have difficulties with accessing such programs due to demographic, cultural, or financial reasons. As such, remote programs are an excellent opportunity for physical therapists to engage with broad audiences to improve effects and impacts, resulting in the provision of services, resources, and information in an easier and faster way [21].

Our study has several limitations. One of the main limitations is the heterogeneity of the sample. We observed that the control group was older and had higher BMIs than those of the telerehabilitation group. However, we also observed that, in relation to lung function, the groups were similar. The low number of participants and the study design, as it was not possible to conduct a randomized clinical trial due to the urgency of reorganizing the rehabilitation service in view of the advance of the COVID-19 pandemic, are also limitations. It is understood that practices may differ across regions and health care providers; hence, a multicenter, prospective randomized study is needed to understand the role of web-based telerehabilitation in a range of clinical settings. Further research is required to determine optimal exercise training modalities and identify strategies for maximizing the long-term benefits in patients with chronic lung diseases.

### Conclusion

This study shows that conducting a telerehabilitation program for patients with chronic lung diseases can ease the deleterious effects of disease progression. Our results indicate that telerehabilitation can be used as a strategy for maintaining functional capacity and improving aspects of quality of life in patients with chronic lung diseases.

## Abbreviations

6MWD: 6-minute walk distance
6MWT: 6-minute walk test
COPD: chronic obstructive pulmonary disease
HRQOL: health-related quality of life
INPTRA: International Network of Physical Therapy Regulatory Authorities
IPF: idiopathic pulmonary fibrosis
ISCMPA: Irmandade da Santa Casa de Misericórdia de Porto Alegre
PRP: pulmonary rehabilitation program
SF-36: 36-item Short Form Health Survey
